# Separate core-shell analysis of urinary stones may influence specific metaphylaxis

**DOI:** 10.1007/s00345-025-06162-7

**Published:** 2026-01-31

**Authors:** F. I. Winterhagen, S. Latz, C. Jacobs, P. Lossin, J. Stein

**Affiliations:** 1 Department of Urology, University Hospital Bonn, Venusberg Campus 1, 53127 Bonn, Germany; 2 Urinary Stone Analysis Center, Urologie Bonn-Rhein-Sieg, Bonn, Germany

**Keywords:** Urolithiasis, Metaphylaxis, Stone analysis, Core-shell analysis

## Abstract

**Purpose:**

The recommendation for urinary stone metaphylaxis is based on urinary stone analysis. It is not known to what extent the separate analysis of the core and shell differ and can therefore possibly influence the metaphylaxis.

**Methods:**

Included were 19,973 stone analyses from 2021 to 2022 using infrared spectroscopy. For mixed stones, the individual components were specified semi-quantitatively in 5% increments. While 62% of the stones were analyzed using a representative sample of the whole stone, a separate core-shell analysis was performed in 38%. Analysis focused on evaluating the distribution of the most common urinary stone minerals.

**Results:**

In 22.9% of cases, difference in analysis between core and shell could be measured, in 13.6%, analyses differed by at least 10%. Here, a clear shift in the distribution of stone types compared to the distribution using a single representative probe could be demonstrated: Calcium oxalate occurred significantly less frequently in this group (50.2% vs. 73.3%), while struvite (5.9% vs. 2.7%) and carbonate apatite (25,2% vs. 12.1%) occurred significantly more frequently. The number of components detected in the stones increased in separate analysis and were even more when core and shell differ. In 4.3%, the main component between core and shell changed.

**Discussion:**

In 13.6% of stones, there were clinically significant differences between core and shell. The change in the main component between core and shell may indicate the stone genesis. Separate core-shell analysis can influence and possibly optimize the metaphylaxis. Especially in the high-risk situation and recurrent stones, a separate core-shell analysis may be beneficial.

## Introduction

The prevalence of urinary stones in countries with a high standard of living is constantly increasing with some regions reporting an increase of over 37% in the last two decades [1–4]. Within five years stones recur in up 26%, recurrence can be reduced to 10% under metaphylaxis [5]. Patients are classified as high-risk stone formers if they have early-onset or recurrent urolithiasis, certain types of stones (e.g., brushite, uric acid, infection stones), associated medical conditions (e.g., CKD, hyperparathyroidism, metabolic disorders), genetic causes, or anatomical abnormalities. Urinary stones contribute potential complications such as acute renal failure or urosepsis as well as chronic kidney disease possible leading to an end stage kidney disease due to the primary conditions causing stone formation or the intervention for stone removal [6, 7].

In high-risk patients, multiple guidelines recommend specific stone metaphylaxis, which can reduce the recurrence risk from 50 to 80% to 10–15% [6, 8, 9]. Strategies of metaphylaxis are based on stone analysis and stone analysis itself can identify a high-risk patient (e.g. struvite, cystine). Therefore, stone analysis is recommended for every first stone event and in case of recurrence under stone metaphylaxis, rapid stone recurrence or stone recurrence after a long stone-free period.

Urinary stones can significantly vary in composition, with calcium oxalate stones representing the predominant form with a frequency of 67–80% [10, 11]. Recent data published in 2022 showed, that in Germany the most common stone mineral calcium oxalate account for 71.4% of all stones [12]. They are followed by those consisting mostly of carbonate apatite with 10.2% (5–16%), and struvite stones, also known as magnesium ammonium phosphate “infection stones” with 2.1% (3–5%). Uric acid stones account for 8.3% (8–13%) and brushite stones for 1.3% while cystine (0.4%) and ammonium urate are rare, each comprising less than 1% of cases.

It is known that stones often are composed of different minerals, and the exact analysis can provide essential information about factors affecting stone formation. When analyzing stones macroscopically, some show a different composition of the core of stones than in the shell, which might provide more information about the history of the stone formation. Only one study was found which included separate core and shell analysis on 10,000 urinary stones. It was shown that 29.5% of these stones showed differences between core and shell. Only weddellite and uric acid stones showed a clear preference of the shell [13]. Recently there has been no data available which analyzed the difference between core and shell. However, there are studies that have demonstrated that urinary stones often exhibit structural and compositional heterogeneity, reflecting distinct phases in their development. This can provide key information for determining metaphylaxis. Morpho-constitutional stone analysis has shown that different regions of a stone may arise from different lithogenic processes and therefore yield important etiological clues that are not captured by a single analysis [14]. Microscopy-based analysis have further demonstrated that many urinary stones consist of multiple mineral phases arranged in concentric or irregular layers [15]. Consequently, evaluating only the overall mineral composition may obscure clinically relevant minority components, especially when these constitute only a small mass fraction but represent an important pathogenic trigger.

Stone metaphylaxis and stone analysis are pivotal aspects in nephrology and urology to prevent urinary stone recurrence and avoid severe complications. Given the rising prevalence of urinary stones and their diverse compositions, an individualized approach of metaphylaxis is imperative. Here, further analysis of the core and shell can provide an important approach to establish precise metaphylaxis and thus further reduce the risk of recurrent stone formation. Therefore, the aim was to analyze the differences in composition between the core and the shell of a stone, to better understand stone formation, improve metaphylaxis, and possibly achieve a reduction in recurrence rates.

## Methods

The study underwent review by the Ethics Committee of the University of Bonn, which concluded that ethical vote is not required (2024-248-BO).

A total of 20,158 urinary stone samples were collected and analyzed at the Urinary Stone Analysis Center in Bonn during the years 2021 and 2022 using Fourier-transform infrared spectroscopy in ATR technique. In the case of mixed stones, the individual mineralss were semiquantitatively indicated in 5% increments. This allowed for a detailed assessment of the varying composition within mixed stones and was personally conducted by the laboratory management after IT-assisted pre-evaluation. This ensured a standardized approach to the analysis of each urinary stone sample.

For explicit requests from the referrer, separate core-shell analyses of the sample were performed. Otherwise, a representative sample of the entire urinary stone was measured. For differentiation of core and shell, first, stone dust is scraped from the surface (shell) and analyzed. For the core analysis, the stone is cut and dust is scraped from the center.

Our statistical analysis focused on the prevalence and distribution of the most common urinary stone minerals. These included whewellite, weddellite, uric acid/uric acid dihydrate, carbonate apatite, brushite, struvite, cystine, and protein. To account for clinical relevance, whewellite and weddellite stones were grouped together as a calcium oxalate.

The main component (mineral) was defined as the constituent that comprises the largest proportion of the stone and defines the type of the stone. In mixed stones, this does not necessarily need to account for 50% of the total mass and may instead be represented by two components of equal prominence. Pure stones are defined as those composed of at least 95% of a single component.

Data analysis was performed using IBM SPSS Statistics v26. Descriptive statistics were calculated for categorical variables, including frequencies and proportions, while continuously coded variables were described with means and standard deviations. Differences were analyzed using chi-square paired tests. An unpaired, two-tailed t-test was used to test statistical significance (*p* < 0.05). Graphics were created with the help of ChatGPT.

## Results

**Fig. 1:**
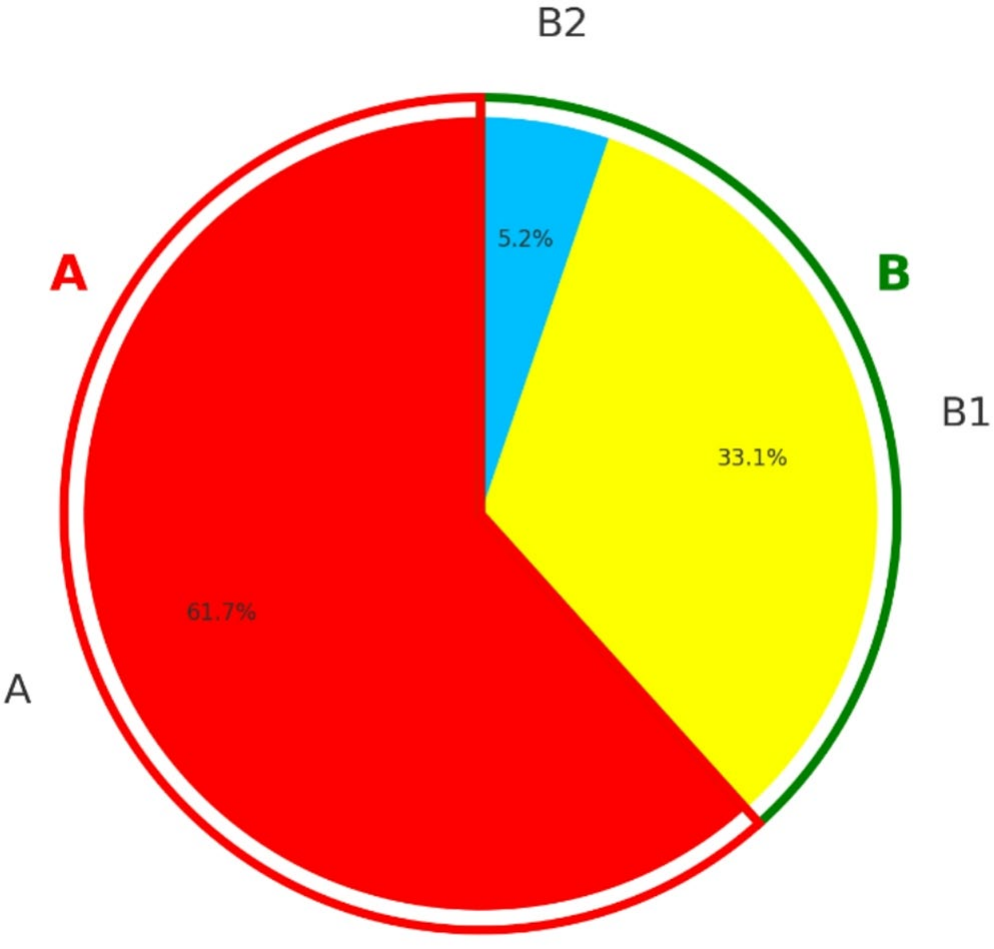
Distribution and naming of groups: In total, 19,973 stone measurements were analyzed, 12,314 (61.7%) were analyzed with single conventional stone analysis (group** A**), 7,658 (38,3%) were analyzed separately in core and shell (group** B**). 6,617 of all stones of group B are said to be equal with a difference in both core and shell < 10% (group B1). 1,041 of all stones differ in their analysis between core and shell with at minimum 10% (group B2)

19,972 of 20,158 stone analyses were included in the statistical evaluation, 186 had to be excluded. Exclusion criteria were that the sum of the stone composition was either greater or less than 100%, minerals other than whewellite, weddellite, uric acid/uric acid dihydrate, carbonate apatite, brushite, struvite, cystine, and protein were present, or that the same mineral appeared twice in the given results. A separate core-shell analysis was performed on 7658 stones (38.3%, Group B), while a conventional single urinary stone analysis was carried out on 12,314 stones (61.7%, group A, Fig. 1).

### Characteristics of the group single analysis (A)

For stones where a single analysis was performed (12,314, group A), 64.3% (7916) were classified as pure when separated according to calcium oxalate, carbonate apatite, uric acid, struvite, cystine, urate and brushite, while 35.7% (4398) were identified as mixed stones.

**Fig. 2:**
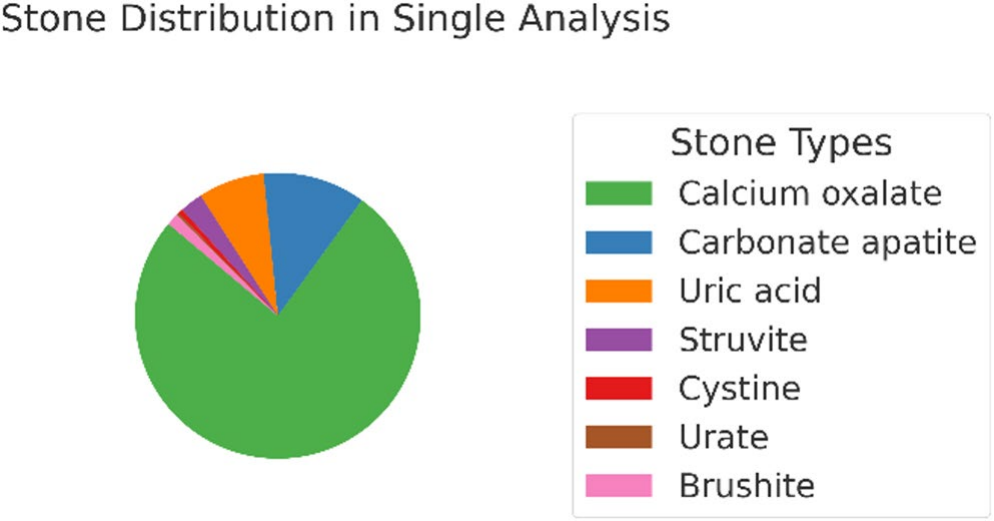
Stone distribution: In single analysis 73.3% of all stones were calcium oxalate, 12.1% carbonate apatite, 7.4% uric acid, 2.7% struvite, 0.6% cystine, 0.3% urate, 1.4% brushite

A total of 73.3% (9021) of all stones were composed of calcium oxalate as the main component, 12.1% (1485) consisted of carbonate apatite, and 7.4% (953) were composed of uric acid. Furthermore, 2.7% (334) were struvite stones, 1.4% (172) were brushite stones, 0.6% (73) were cystine stones, and 0.3% each were classified as protein or urate stones. In 0.9% (110) of all stones, calcium oxalate and carbonate apatite were present in equal proportions, while other mixed main components together accounted for 2.4% (Table 1; Fig. 2).

**Table 1:** Main component of stones after single analysis (A): A main component is the one that constitutes the largest percentage of the stone; in mixed stones, it does not necessarily have to be 50% of the total mass and can also be determined by two equally frequent components

Stone Type (main component of stone)	Number	%
Uric Acid	953	7.4
Calcium Oxalate	9021	73.3
Carbonate Apatite	1485	12.1
Struvite	334	2.7
Cystine	73	0.6
Protein	41	0.3
Urate	39	0.3
Brushite	172	1.4
Calcium Oxalate and Carbonate Apatite	110	0.9
Others > 1 main component	86	0.7

### Characteristics of the group core-shell analysis (B)

For stones where a separate analysis of core and shell was performed (7658, Group B), 61.9% (4743) of stones were classified as pure, while 38.1% (2915) were identified as mixed stones consisting of more than one component.

**Fig. 3:**
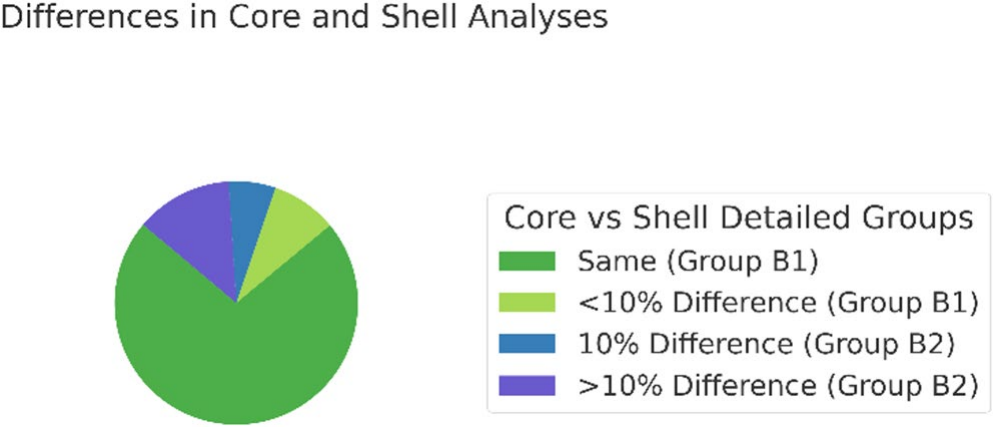
Difference between core and shell: In 77.1% of all core and shell analysis results were same, in 22.9% of cases they differ. 9.3% of all stones differ less than 10%, 6.8% differ 10% and 6.8% differ more than 10%. Those stones differing less than 10% are said to be group B1 and those differing at least 10% are said to be group B2

In total, 65.3% (5683) of all core-shell comparisons yielded identical results. Varied findings in the separate analysis accounted for 34.7% (2657), with 25.8% (1975) of these differing by at least 10% (Fig. 3). When whewellite and weddellite were combined as calcium oxalate, 77.1% (5903) of analyses were equal, and divergent results were observed in 22.9% (1761) of cases, with 13.6% (1041) showing a difference of at least 10%. This subgroup was subsequently defined as Group B2.

In total, 86.4% of stones in which whewellite and weddellite were combined as calcium oxalate were equal or differed by a maximum of 5% between core and shell; this subgroup was defined as Group B1. Further subgroup analysis showed that 3.3% (256) differed by more than 20% (Table 2).

**Table 2:** Core and shell were analyzed to determine if they differ in their composition

Core and Shell analysis	Number	Percent
Equal	5,903	77.1
Unequal	1755	22.9
- Difference < 10%	714	9.3
- Difference ≥ 10%	1047	13.6
Equal = Difference < 10%	6617	86.4
Unequal ≥ 10%	1041	13.6
- Difference 10%	504	6.8
- Difference 15–20%	264	3.4
- Difference > 20%	250	3.3
- Difference 100%	6	0.1

22.9% of B showed differences in composition between the core and shell, with a difference of at minimum 10% (B2) in 13.6% of cases. A difference of 10% could be measured in 6.8% of cases, 15–20% difference in 3.4%, and over 20% difference in 3.3%. Equal measurements in core and shell or a difference of 5% (B1) represented 86.4% of all stones

In 3.9% (300) of all core-shell analyses, new components in the core that were not present in the shell, or vice versa, with at least 10% of the stone mass were detected. This corresponded to 28.8% of all cases in which the core and shell differed.

#### Stone composition

For analysis and comparison of Group B, the mean value of core and shell composition was calculated. For comparability, in Group B2 the mean percentage for each mineral (component) in the core and shell was calculated (e.g., brushite represented 2.3% of all core analyses and 2.5% of all shell analyses; therefore, brushite was considered to represent 2.4% of all core-shell analyses in Group B). However, the overall mineral composition of the entire stone was influenced by different core-shell volume ratios (e.g. very thick shell and small core or vice versa). For the sake of comparability, mean values were reported.

Stones were classified according to their dominant component; for example, a stone composed predominantly of calcium oxalate was classified as a calcium oxalate stone.

When analyzing cases in which core and shell were measured separately (Group B), calcium oxalate was present in the core in 75.1% (5,752) and in the shell in 74.8% (5,726) of cases (Table 3). In the subgroup in which core and shell had identical composition (Group B1), calcium oxalate represented 78.9% (5,520) of cases. In stones with different core and shell composition (Group B2), calcium oxalate represented 50.9% (532) of core samples and 49.9% (512) of shell samples. Calcium oxalate occurred significantly more frequently in Group A than in Group B (*p* = 0.01). Within Group B, it was more frequent than in Group B1 (*p* < 0.00) or Group B2 (*p* < 0.001). There was no significant difference between core and shell composition (*p* > 0.382).

In Group B, carbonate apatite was detected in the core in 10.0% (764) and in the shell in 9.8% (752) of cases. In Group B1, it was present in 7.5% (494) of stones. In Group B2, carbonate apatite represented 25.8% (270) of core samples and 24.5% (256) of shell samples. It was observed significantly more often in Group A compared with Group B (*p* < 0.00). Significant differences were noted between Group B and Group B1 (B < B1), as well as between Group B and Group B2 (B < B2) and between Group B1 and Group B2 (*p* < 0.00). It was most common in Group B2 (*p* < 0.00). There was no significant difference between core and shell composition (*p* = 0.59).

**Table 3:** Stone type of core and shell

Stone type	Core (%)	Shell (%)	Single analysis	Literature
Uric acid (all core and shell (B, 7658))	8.5^#+^	8.7^#+^	7.4	8
- Equal (B1 6,617)	8.3’^#^	8.3’^#^		
- Unequal min. 10% (B2 1,041)	9.7^+#^	11.1^+#^		
Calcium oxalate (all core and shell) B	75.1*^+^’	74.8*^+^’	73.3	67–80
- Equal	78.9’^#^	78.9’^#^		
- Unequal min. 10%	50.9^+#^	49.9^+#^		
Carbonate apatite (all core and shell)	10.0*^+^’	9.8*^+^’	12.1	5–16
- Equal	7.5’^#^	7.5’^#^		
- Unequal min. 10%	25.8’^#+^	24.5’^#+^		
Struvite (all core and shell)	2.2*^+^’	2.3*^+^’	2.7	3–5
- Equal	1.7’^#^	1.7’^#^		
- Unequal min. 10%	5.4^+^	6.3^+^		
Cystine (all core and shell)	0.5	0.5	0.6	0.4
- Equal	0.5	0.5		
- Unequal min. 10%	0.1	0.1		
Brushite (all core and shell)	1.6	1.7	1.4	0.9
- Equal	1.5	1.5		
- Unequal min. 10%	2.3	2.5		
Calcium oxalate + Carbonate apatite	0.9*^+^	1.1*^+^	0.9	
- Equal	1.5^*#^’	1.5^*#^’		
- Unequal min. 10%	3.4^+^	4.8^+^		

First, percentage of all core-shell analysis (B) which is represented by the mentioned type of stone (7,658). In sub analysis, listed are those cases where core and shell differ in their composition at a minimum of 10% (unequal, 1,041) and differ not (equal, 6,617). *Analysis differs significantly from single analysis (A), ^#^ to B, ^+^to B1, ’ to B2, ^^^between core and shell. In no case could a significant change in the frequency of occurrence between core and shell be measured

Uric acid was detected in the core in 8.5% (652) and in the shell in 8.7% (667) of cases. In subgroup B1, it was observed in 8.3% (551) of cases. In B2, uric acid represented 9.7% (101) of core samples and 11.1% (116) of shell samples. Uric acid did not occur significantly more frequently in all core-shell analyses (B) compared to single analysis (*p* = 0.051), but was more frequent in B1 than in B (*p* = 0.02) and less frequent in B2 (*p* = 0.04). There was no significant difference between core and shell in B (*p* = 0.52) and no significant difference between B1 and B2 (*p* = 0.65).

For stones in which a difference between core and shell was present (B2), struvite represented 5.4% (57) of core samples and 6.3% (66) of shell samples, whereas in all core-shell analyses (B) struvite represented 2.2% (168) of all cores and 2.3% (177) of all shells. In B1, struvite accounted for 1.7% (111) of cases. Compared to single analysis, struvite occurred significantly less frequently in B (*p* = 0.049). In B1 it occurred significantly less frequently than in B (*p* = 0.02), and in B2 it occurred significantly more frequently (*p* < 0.001). Statistically, there was no difference between core and shell (*p* > 0.457).

Cystine was detected in 0.5% (35) of cases in B and in 0.5% (34) of cases in B1. In B2, cystine was present in both core and shell in 0.1% (1). Cystine did not differ significantly in any subgroup (*p* > 0.029).

In B2, brushite represented 2.3% (24) of cores and 2.5% (26) of shells, whereas in B it represented 1.6% (126) of cores and 1.7% (128) of shells. In B1, brushite accounted for 1.5% (102). In single analysis, brushite did not occur significantly differently compared to B, B1, or B2, nor were there significant differences between B1 and B2. There was no significant difference between core and shell (*p* > 0.886).

Stones in which calcium oxalate and calcium phosphate were identified as equivalent main components represented 0.9% (68) of all cores in B and 1.1% (86) of shells. In B1 they were present in 1.5% (32) of stones, whereas in B2 they accounted for 3.4% (36) of cores and 4.8% (50) of shells. These stones appeared significantly more often in single analysis than in B (*p* < 0.00), more often in B than in B1 (*p* < 0.00), and more often in B2 than in B1 (*p* < 0.00). There was no significant difference between core and shell (*p* = 0.152).

**Fig. 4:**
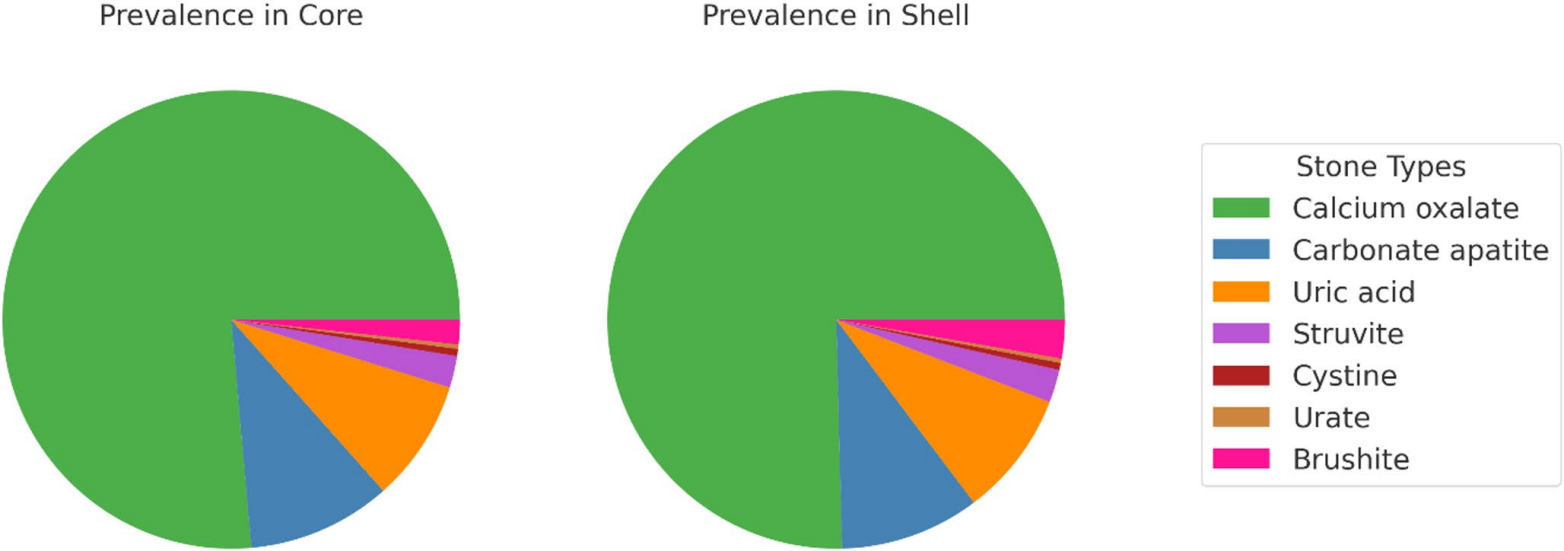
Prevalence of stone types in Core (a) and Shell (b):** a** In separate analysis 75.1% of all were Calcium oxalate, 10.0% Carbonate apatite, 8.5% Uric acid, 2.2% Struvite, 0.5% Cystine, 0.3% Urate, 1.6% Brushite.** b** In separate analysis 74.8% of all stones were Calcium oxalate, 9.8% Carbonate apatite, 8.7% Uric acid, 2.3% Struvite, 0.5% Cystine, 0.2% Urate, 21.7% Brushite

In 95.7% (7,329) of cases, the main component (largest stone proportion) remained the same in the core and shell, while in 4.3% (329) of cases a shift was observed (Fig. 4). In 103 cases (1.3%), a shift occurred from calcium oxalate in the core toward carbonate apatite in the shell, and vice versa in 64 cases (0.8%). In 39 cases (0.5%), the shell shifted to uric acid. In total, 23 cores shifted to struvite as the main component (2.3%).

### Comparison of the number of components between the groups

The number of components was examined in more detail, including the 5% threshold. In the single-analysis group (A), 64.3% (7,916) consisted of one component, 35.3% (4,224) consisted of two components, and 1.4% (175) consisted of three components.

In Group B, 14.1% (1,081) of stones were composed of one component, 31.1% (4,682) contained two components, 24.1% (1,843) contained three components, and 0.7% (52) contained four components.

In Group B1, 16.0% (1,080) of stones were composed of one component, 65.0% (4,297) contained two components, 18.6% (1,230) contained three components, and 0.1% (6) contained four components.

In Group B2, 18.4% (385) of stones contained two components, 22.3% (613) contained three components, and 1.1% (46) contained four components. Additionally, 21.4% (224) of samples in B2 exhibited different components in the core and shell.

**Fig. 5:**
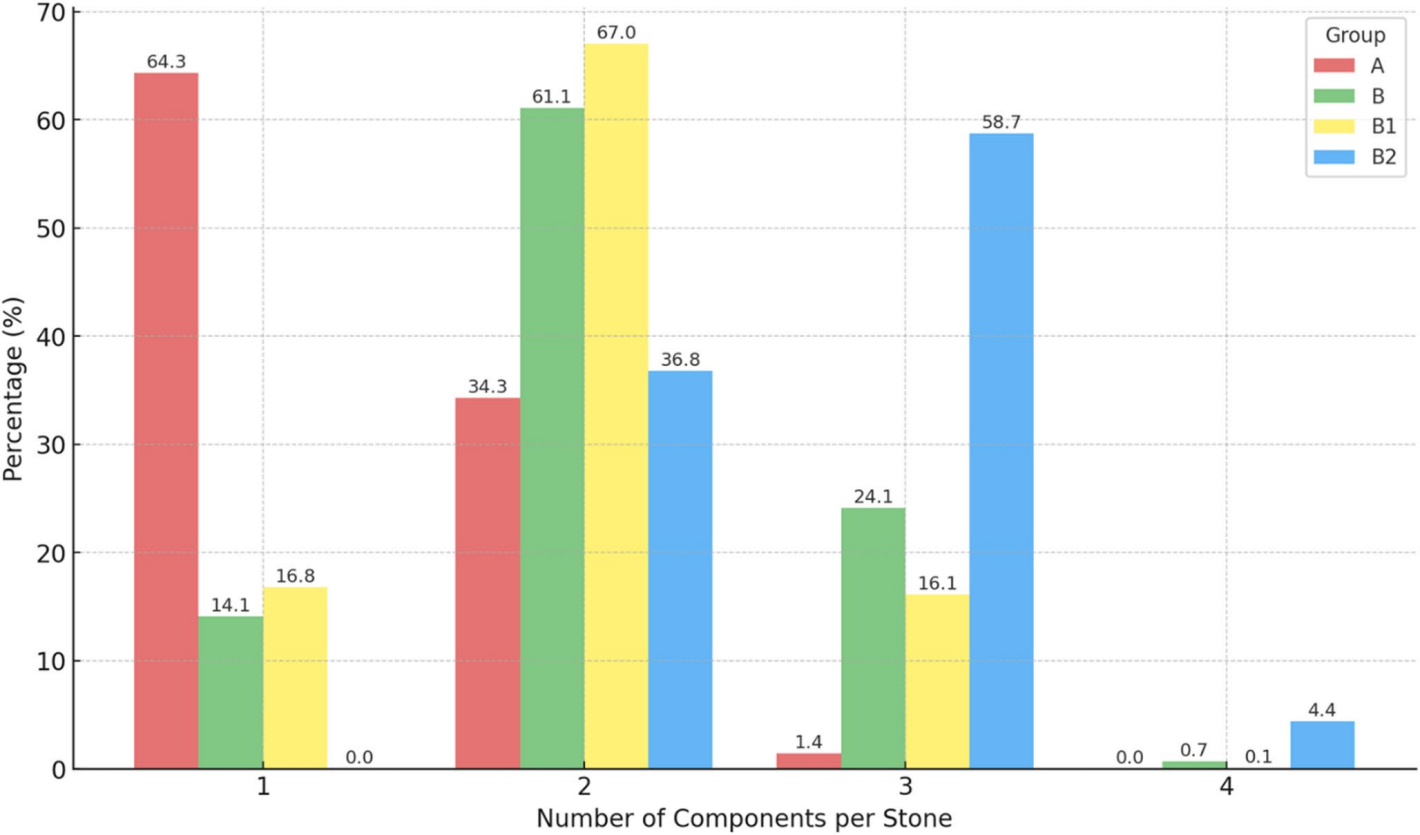
Distribution of component counts across groups [%]. In A, 64.3% show one, 34.3% show two and 1.4% show 3 components. In B, 14.1% show 1, 61.1% show 2 and 24.1% show 3 and 0.7 show 4 components. In B1, 16,8% show one, 67% show two, 16.1% show 3 and 0.1 show 4 compomnents. In B2 36.8% show two, 58.7% show three and 4.4% show 4 components. 5% counts were not excluded

There was a significant difference in the number of components across all groups (A vs. B, A vs. B1, B1 vs. B2; *p* < 0.00) (Fig. 5).

### Occurrence of different minerals according to normalized prevalence

When comparing the normalized prevalence per component of stone constituents between groups A, B, B1, and B2, several clear patterns emerged (Table 4). After normalization for the number of components per stone, uric acid prevalence was comparable across all groups (4.6–5.6%), indicating that previously observed differences were largely attributable to variations in stone complexity. Differences were mostly absent or moderate; B2 exhibited a slightly higher prevalence than B, while B1 and A showed similarly high values (Table 5).

Calcium oxalate remained the most frequent component overall but was markedly lower in B2 (22.15%) compared with A (53.1%), B1 (47.3%), and B (40.9%). It showed extremely significant differences in almost all pairwise comparisons, with B1 and A consistently higher than B, and especially higher than B2, which had the lowest prevalence.

In contrast, calcium phosphate was proportionally higher in B2 (11.16%) and A (8.74%) than in B (5.40%) and B1 (4.23%). It was significantly more frequent in B2 in many comparisons, particularly against B1 and B.

Struvite was most frequent in B2 (2.61%), followed by A (1.97%), B (1.23%), and B1 (0.95%), with B2 demonstrating consistently and often highly significant higher prevalence compared with all other groups.

Brushite proportions were similar across groups (0.87–1.06%), with a slight peak in B2. Most differences were not significant, except for a slightly higher prevalence in B2 compared with A.

Cystine was rare in all groups (< 0.5%), with the lowest proportion in B2 (0.04%), and no significant differences were observed.

**Table 4:** Normalized prevalence per component (%) of stone constituents by Group. Normalized prevalence is calculated as the proportion of stones containing the respective component, adjusted for the number of components per stone, allowing for direct comparison between groups with different stone complexities

Stone	A	B	B1	B2
Uric acid	5.61	4.65	5.62	4.62
Calcium oxalate	53.1	40.89	47.27	22.15
Carbonate apatite	8.74	5.4	4.23	11.16
Struvite	1.97	1.23	0.95	2.61
Brushite	1.01	0.9	0.87	1.06
Cystine	0.43	0.25	0.29	0.04

## Discussion

### Prevalence of mineral comparing single analysis and core-shell analysis in general

According to current data, calcium oxalate stones are the most common stones, followed by carbonate apatite, uric acid stones, struvite, and brushite. Cystine stones are known to be rare. Among the stones for which a single conventional analysis was performed, the frequency of occurrence corresponds to the literature with calcium oxalate representing 73.3% of all stones, carbonate apatite 12.1%, uric acid 7.4%, struvite 2.7 and brushite with 1.4%. The mixed stones containing calcium oxalate and carbonate apatite equally represent 0.9% of all stones.

This ranking regarding the prevalentce of stones is similar when core and shell are separately analyzed (B). In this group calcium oxalate represents 75% of all stones, carbonate apatite 10%, uric acid 8.6%, struvite 2.3 and brushite 1.7%. The mixed stones containing calcium oxalate and carbonate apatite equally represent 1% of all stones.

But significant differences in the prevalence of stones in those being analyzed in one analysis using a representative probe (A) compared to separate analysis of core and shell (B) become apparent. Calcium phosphate is most present in single analysis and does appear even less in those stones showing differences in core and shell. Here, no benefit can be seen when performing different analysis of core and shell.

Carbonate apatite and struvite are significantly more present in B2. Uric acid occurs equally in single analysis and B but is significantly most often represented in B1. This shows that single analysis detects uric acid more often and might be missed during single analysis. Struvite is slightly more often present in single analysis but it is highly significant more often present in B2. Struvite stones tend to be formed in presence of urinary tract infection, which can be caused by and induce further stone formation. It might be important to further analyze stones containing struvite to get knowledge of the real stone composition.

Cystine did not appear significantly different in any since cystine stone is known to be particularly pure. Reason for this might be the well known genetic origin of stone formation, those patients usually have regular follow up visits.

### Prevalence of stone composition comparing core-shell analysis for B1 and B2

In B2 there was a significant shift in prevalence of the stone composition. Carbonate apatite and struvite occur significantly more frequently in B2 than in B1, brushite and uric acid occur more frequently in B2, but not on significant level. Calcium oxalate is presented significantly more frequently in B1, Cystine is more prevalent in B1, however it is not statistically relevant. This can be in part be explained by the stone characteristics and the genesis of the stone development. Cystine stone is known to be mostly pure, however the development of a different shell an be potentially caused by specific stone metaphylaxis. A urinary tract infection can be caused by the presence of a stone, in this case, struvite and carbonate apatite as so called “infectious stones” may crystallize what makes it more likely to be part of a mixed stone.

Overall, B2 exhibited the lowest normalized calcium oxalate proportion but the highest relative prevalence of carbonate apatite, struvite, and brushite, suggesting a more heterogeneous and potentially infection-related stone composition. For carbonate apatite and struvite a separate analysis of core and shell seems to have a clear benefit, for uric acid and brushite it should be considered.

Concerning the number of stone components more components could be detected when separate analysis was performed (B), interestingly, those stones showing difference in core and shell (B2) are built up of more components.

**Table 5:** Summary of statistically significant comparisons (Chi² test) for each substance across A, B, B1, and B2, with the group showing higher normalized prevalence per component indicated in parentheses. Whenever single analysis is relevant it is in bolt letters

Substance	Significant comparisons
Uric acid	A vs. B1 (**B1**); A vs. B2 (**B2**); B vs. B1 (B1); B vs. B2 (**B2**); B1 vs. B2 (B1)
	relevance and recommendation for separate analysis: should be considered
Calcium oxalate	A vs. B (**B**); A vs. B1 (B1); A vs. B2 (A); B vs. B1 (B1); B vs. B2 (B); B1 vs. B2 (B1)
	relevance and recommendation for separate analysis: no
Carbonate apatite	A vs. B (A); A vs. B1 (A); A vs. B2 (**B2**); B vs. B1 (B); B vs. B2 (**B2**); B1 vs. B2 (**B2**)
	relevance and recommendation for separate analysis: yes
Struvite	A vs. B (A); A vs. B1 (A); A vs. B2 (**B2**); B vs. B1 (B); B vs. B2 (**B2**); B1 vs. B2 (**B2**)
	relevance and recommendation for separate analysis: yes
Brushite	A vs. B2 (**B2**)
	relevance and recommendation for separate analysis: should be considered
Cystine	None
	relevance and recommendation for separate analysis: no

### Overall discussion

These data show that with single analyses, there is a risk of underdiagnosing specific stone components, which could lead to misguided specific metaphylaxis. Therefore, if results of single analysis identify additional urinary stone components that contradict the basic principles of metaphylaxis, special care should be taken. In conclusion, it can be said, that for carbonate apatite and struvite stones, separate core-shell analysis should be recommended, as well as for uric acid and brushite. However, for those patients suffering from stone recurrence although they have metaphylaxis separate analysis also in other stones might be helpful to adjust metaphylaxis.

For example: A stone containing calcium oxalate, carbonate apatite and struvite. The specific metaphylaxis for calcium oxalate stones is based on alkali citrates. However, these are potentially lithogenic for carbonate apatite and struvite due to their urinary alkalinizing effect. In this case, citrate substitution should be considered cautiously and depending on the urine collection analysis (e.g. in the case of hypocitraturia). On the contrary, one might consider lowering urine pH with methionine and deliberately forgoing citrate substitution. Since bacteria-associated stones and components are prone to rapid recurrences, antibiotic prophylaxis may also be necessary even with smaller amounts of struvite or carbonate apatite. Moreover, the finding of such a stone composition should warrant increased awareness regarding phosphate lithogenesis. Regular urine culture checks are advisable, and it would also be advisable to look more closely for predisposing factors for urinary tract infections, such as bladder emptying disorders, urinary reflux or urinary tract obstructions.

However, to perform a core-shell analysis, both the core and the shell must be present in the sample sent in. This means that a representative piece of material containing both components must be available or clearly labeled parts of the stone must be saved. The size of the collected stone samples is primarily determined by the surgical procedure. In case of percutaneous nephrolitholapaxy larger fragments are typically retrieved, allowing for a core-shell analysis. However, with retrograde intrarenal surgery (RIRS), especially in the era of dusting, sufficiently large fragments that include both the core and shell are usually not obtained. Often only small samples are extracted, usually the core, as these are the fragments that remain at the end of the dusting procedure. A core shell analysis is feasible with samples that are at least 4–5 mm in size. It must be possible to split the stone to identify and separately analyze samples from the core and the shell.

## Conclusion

To conclude, one should consider a separate core-shell analysis for stones containing carbonate apatite and struvite in the first place. It might not be necessary in the first place for calcium oxalate and cystine. With single analyses, there is a risk of misinterpreting important components of the stone and thus the genesis of the stones. Therefore, a potentially effect of core-shell analysis on metaphylaxis strategies is assumed. At least in high-risk stone formers and especially in patients with stone recurrence despite medication-based metaphylaxis, core-shell analysis can potentially enhance diagnostic value and help to optimize and individualize specific and stone metaphylaxis.

## Data Availability

No datasets were generated or analysed during the current study.

## Abbreviations

PNL: Percutane nephrolitholapaxy
URS: Ureterorenoscopy
